# Anti-c-Met monoclonal antibody ABT-700 breaks oncogene addiction in tumors with *MET* amplification

**DOI:** 10.1186/s12885-016-2138-z

**Published:** 2016-02-16

**Authors:** Jieyi Wang, Liliane Goetsch, Lora Tucker, Qian Zhang, Alexandra Gonzalez, Kedar S. Vaidya, Anatol Oleksijew, Erwin Boghaert, Minghao Song, Irina Sokolova, Ekaterina Pestova, Mark Anderson, William N. Pappano, Peter Ansell, Anahita Bhathena, Louie Naumovski, Nathalie Corvaia, Edward B. Reilly

**Affiliations:** AbbVie, North Chicago, IL USA; IRPF, Centre d’Immunologie Pierre Fabre 5, Av Napoléon III, F-74164 Saint-Julien-en-Genevois, France; Abbott Molecular, Des Plaines, IL USA; AbbVie Biotherapeutics, 1500 Seaport Blvd., Redwood City, CA 94063 USA

**Keywords:** *MET*, c-Met, *MET* amplification, oncogene addiction, ABT-700

## Abstract

**Background:**

c-Met is the receptor tyrosine kinase for hepatocyte growth factor (HGF) encoded by the *MET* proto-oncogene. Aberrant activation of c-Met resulting from *MET* amplification and c-Met overexpression is associated with poor clinical outcome in multiple malignancies underscoring the importance of c-Met signaling in cancer progression. Several c-Met inhibitors have advanced to the clinic; however, the development of inhibitory c-Met-directed therapeutic antibodies has been hampered by inherent agonistic activity.

**Method:**

We generated and tested a bivalent anti-c-Met monoclonal antibody ABT-700 in vitro for binding potency and antagonistic activity and in vivo for antitumor efficacy in human tumor xenografts. Human cancer cell lines and gastric cancer tissue microarrays were examined for MET amplification by fluorescence in situ hybridization (FISH).

**Results:**

ABT-700 exhibits a distinctive ability to block both HGF-independent constitutive c-Met signaling and HGF-dependent activation of c-Met. Cancer cells addicted to the constitutively activated c-Met signaling driven by *MET* amplification undergo apoptosis upon exposure to ABT-700. ABT-700 induces tumor regression and tumor growth delay in preclinical tumor models of gastric and lung cancers harboring amplified *MET*. ABT-700 in combination with chemotherapeutics also shows additive antitumor effect. Amplification of *MET* in human cancer tissues can be identified by FISH.

**Conclusions:**

The preclinical attributes of ABT-700 in blocking c-Met signaling, inducing apoptosis and suppressing tumor growth in cancers with amplified *MET* provide rationale for examining its potential clinical utility for the treatment of cancers harboring *MET* amplification.

**Electronic supplementary material:**

The online version of this article (doi:10.1186/s12885-016-2138-z) contains supplementary material, which is available to authorized users.

## Background

Amplification of the *MET* gene, with consequent c-Met receptor tyrosine kinase (RTK) overexpression and constitutive kinase activation, is an oncogenic driver in multiple malignancies [1–4]. Unlike other oncogene RTKs including the ERBB family members which have been clinically targeted with therapeutic antibodies, the development of inhibitory c-Met-directed therapeutic antibodies has been challenging [3, 5–7]. Binding of c-Met by HGF or overexpression of c-Met on cell surface independent of ligand induces dimerization and activation of the receptor tyrosine kinase [2, 8]. Previously reported bivalent antibodies generated against c-Met often mimic HGF, promoting productive dimerization and activation of c-Met [9, 10]. The engineered monovalent antibody, MetMAb (onartuzumab), avoids this agonistic activity [11] but the monovalent nature of MetMAb may limit the scope of its activity to HGF-dependent c-Met signaling, similar to the HGF-binding antibodies [6].

ABT-700 is a bivalent humanized IgG1 that displays distinctive properties compared to other c-Met-targeting antibodies. ABT-700 binds cellular c-Met and disrupts its productive dimerization and activation induced by HGF or by the high density of c-Met on the cell surface independent of ligand. We hypothesize that ABT-700 might be effective in treating cancers harboring amplified *MET* and focused preclinical studies to assess its antitumor activity in models driven by *MET* amplification. These findings provide scientific rationale for the clinical activity observed in patients with *MET* amplified tumors following treatment with ABT-700.

## Methods

### Antibodies, reagents and cell culture

ABT-700, an anti-human c-Met antibody derived from the mAb 224G11 [12] was produced in a stable CHO line. Fab and F(ab)’2 of mAb224G11 (ABT-700) were generated by digestion with papain or pepsin as described in the literature [13]. Control human IgG was purchased from Sigma (I4506). 5D5 mouse anti-human c-Met antibody, the parental bivalent antibody from which the single armed antibody onartuzumab was derived, was purified from hybridoma supernatant (ATCC #HB11895). The anti-c-Met antibody, LY2875358, was expressed in and purified from HEK293 cells using amino acid sequences derived from published patent application US201012936. The c-Met tyrosine kinase inhibitor, PF-4217903, was purchased from Selleck (Catalog No.S1094). Recombinant human c-Met extracellular domain with a histidine tag (rh-c-Met ECD-6His) was expressed in and purified from HEK293 cells. HGF was purchased from R&D (rhHGF, #294-HGN/CF). The tumor cell lines A549 (ATCC #CCL-185), EBC1 (JCRB #0820), Hs746T (ATCC #HTB-135), and OE33 (Sigma #96070808) were maintained in DMEM (Gibco-Invitrogen cat. No. 11995) supplemented with 10 % fetal bovine serum (FBS) (HyClone SH30070.03). IM95 (JCRB #1075) were also maintained in DMEM, 10 % FBS with 10 mg/L insulin. SNU5 (ATCC #CRL-5973), NCI-H441 (ATCC #HTB-174), NCI-H1993 (ATCC #CRL-5909), MKN45 (JCRB 0245), SNU620 (KCLB #00620), and SNU638 (KCLB #00638) were cultured in RPMI-1640 (Gibco-Invitrogen, cat. No. 11875) supplemented with 10% FBS. MCF7 cells (ATCC HTB-22) were infected with control lentivirus or lentivirus containing human c-Met cDNA in pLVX-IRES-puro vector (Clontech). Stable clones overexpressing human c-Met protein indicated by Western Blot and FACS were isolated. These cells were grown in DMEM (Gibco-Invitrogen cat. No. 11995) supplemented with 10 % fetal bovine serum (FBS) (HyClone SH30070.03) and 2 μg/mL puromycin (Sigma). All cell lines were expanded in culture upon receipt and cryopreserved to provide cells at similar stage passages for all subsequent experiments. For cell lines not authenticated in the 6 months before use, c-Met expression was confirmed by FACS analysis. Information of additional cell lines is summarized in Additional file 1: Table S1.

### Binding ELISA

96-well plates (Costar #3369) were coated with 100 μL/well of mouse anti-His antibody (Invitrogen #37-2900) at 1 μg/mL in PBS pH7.4 at 4 °C overnight, and then blocked using Superblock (Pierce, #37535) for one hour at room temperature. Plates were washed 4 times with PBST and then incubated with 100 μL of recombinant human c-Met extracellular domain (rh-c-Met ECD-6His) at 2 μg/mL in 10 % Superblock in PBST for 1 h at room temperature. Plates were washed 4 times with PBST and then incubated with ABT-700 or control human IgG in serial dilutions in 10 % Superblock in triplicate wells at room temperature for 1 h. Plates were washed 4 times with PBST and then incubated with 100 μL of 1:15,000 goat anti-human IgG-HRP (Thermo-scientific Pierce, Cat#31412) at room temperature for 1 h. Plates were washed 4 times in PBST and 100 μL of TMB (Pierce, #34028) was added to each well and incubated at room temperature until color developed (approximately 10 min). Reactions were stopped by addition of 2N sulfuric acid (Mallinckrodt chemicals, Cat#H381-05) and optical density (OD) was read at 450 nm.

### FACS analysis

For cellular c-Met binding studies, cells were harvested from flasks when approximately 80 % confluent using Cell Dissociation Buffer (Invitrogen #13151-014 or #13150-016). Cell viability was checked by trypan blue staining to ensure >90 % live cells. Cells were washed once in PBS/1 % FBS (FACS buffer) then resuspended at 1.5-2.5 × 10^6^ cells/mL in FACS buffer. Cells were added to a round bottom 96-well plate (BD Falcon #3910) at 100 μL/well. Ten μL of a 10x concentration of ABT-700 or controls in duplicate wells was added and plates were incubated at 4 °C for four hours. Wells were washed twice with FACS buffer then resuspended in 50 μL of 1:500 anti-human IgG Ab (AlexaFluor 488, Invitrogen #11013) diluted in FACS buffer. Plates were incubated at 4°C for one hour then washed twice with FACS buffer. Cells were then resuspended in 100 μL of PBS/1 % formaldehyde and analyzed on a Becton Dickinson LSRII flow cytometer. FACS binding studies were performed for each cell line in at least two independent experiments.

For Annexin V apoptosis detection, tumor cells were plated at 300,000 cells/ well in 12-well dishes in 2 ml serum-free media (RPMI, 0.1 % BSA). Cells were incubated overnight at 37 °C, 5 % CO_2._ Cells were treated with control hIgG (Sigma I4506) and ABT-700 at 10 μg/ml for 24 h. Cells were transferred from 12-well plate into 1.5 ml microcentrifuge tubes, pelleted, and washed with cold PBS**.** Cells were resuspended in 0.1 ml 1X Binding Buffer provided in kit (BD Pharmingen kit cat# 556547). 5 μl of FITC Annexin V and 5 μl propidium iodide (PI) were added and cells were incubated in the dark for 15 min. 400 μl of 1X Binding Buffer was added and cells were analyzed on a Becton Dickinson LSRII flow cytometer within one hour.

### Determination of cellular c-Met phosphorylation and total level

A549 cells were plated at 40,000 per well in 96-well plate in growth media. Twenty four hours later, cells were pretreated with antibodies in duplicate wells for one hour at 37 °C, and then stimulated with HGF for 10 min at 37 °C. 1 nM (~100 ng/mL) HGF was used to stimulate c-Met as described in the literature [11]. For SNU5 cells that have constitutively phosphorylated c-Met, the cells were plated at 20,000 per well in 96-well V-bottom plate in serum free medium. Twenty four hours later, cells were treated with antibodies in duplicate wells for six hours at 37 °C. Media were then removed and cells were lysed with 100 (for A549) or 150 (for SNU5) μL/well of Cell Lysis Buffer (Cell Signaling Technology #9803) supplemented with protease inhibitor tablet (Roche #11714900). ELISA capture plates were generated by pre-coating wells with 100 μL of an anti-c-Met antibody (R&D systems, # MAB3581) at 2 μg/mL) at 4 °C overnight, followed by blocking with 200 μL/well PBS/1 % BSA treatment for one hour at room temperature, and washed three times in PBST. Cell lysates were added to capture plates and incubated at 4°C overnight. Plates were washed 3 times in PBST, and incubated with anti-phospho-tyrosine 4G10-HRP conjugate (Millipore #16-105; 1:1000 diluted in PBST + 1 %BSA) for 2 h at room temperature. To determine total c-Met, secondary anti-c-Met HRP conjugate was used. Plates were washed 3 times in PBST and 100 μL of TMB was added to each well and incubated at room temperature until color developed. Reactions were stopped by addition of 100 μL/well 2N sulfuric acid, and the OD was read at 450 nm. These studies were performed for each cell line in at least two independent experiments.

### Western blot analysis

Cells were plated at 300,000 per well in 12-well tissue culture plates and were incubated overnight in growth media. Cells were incubated with ABT-700 or control for the time points as indicated at 37 °C. Cells were then lysed with 100 μL/well of 2X LDS NuPAGE sample buffer (Invitrogen NP0007) with reducing reagent (Invitrogen NP0009). Cell lysates were resolved by SDS-PAGE using 4-12 % Bis-Tris NuPAGE gels (Invitrogen NP0322) and transferred to PVDF membranes (Millipore Immobilon-FL # IPFL07810). Blots were blocked with Odyssey Blocking Buffer (LI-COR # 927-40000) for one hour at room temperature, washed three times with PBST, and then incubated overnight with appropriate primary antibodies at 4 °C. Following overnight incubation with primary antibodies, blots were washed three times with PBST for ten minutes, and then incubated with either AlexaFluor680 goat anti-rabbit IgG (Invitrogen A21109, 1:10000) or goat anti-mouse IRDye 800CW (Odyssey # 926–32210, 1:5000) for one hour at room temperature. Blots were then washed three times with PBST, and visualized by scanning using an LI-COR Instrument Odyssey (Model # 9120). Primary antibodies included mouse anti-c-Met (Cell Signaling # 3148 or # 3127), rabbit anti-phospho-Y1234 c-Met (Cell Signaling Technology, # 3077), phosphor-PLCr (Cell Signaling #2821) , phosphor-Erk (Invitrogen #44680G), phosphor-Bad (Cell Signaling #9291), total bad (Cell Signaling #9292), Bim (Cell Signaling #2819), Bcl-xL (BD #51-6646GR), cytochrome C (BD Pharmingen Cat 556433), cleaved PARP (Epitomic #1074-1), and mouse anti-actin (Sigma, # A5441). At least two independent experiments were carried out for each cell line.

### Cytochrome C release assay

SNU5 cells (4×10^6^) were treated with control hIgG or ABT-700 for 24 h. Cells were washed in PBS, pelleted and resuspended in 50–100 μL Digitonin Lysis buffer (75 mM NaCl, 8 mM Na2HPO4, 1 mM NaH2PO4, 1 mM EDTA, 350 μg/mL digitonin, and 250 mM sucrose) by pipetting up and down, and incubated for 30 s. Cells were pelleted at high speed in microcentrifuge for 1 min, supernatant (cytosolic fraction) was collected and 2x NuPAGE sample buffer was added for Western analysis. Pellets (organelle-containing membrane fraction) were washed in cold PBS 3 times, and lysed by sonication in 1x NuPAGE sample buffer for Western analysis. The study was performed in two independent experiments.

### Proliferation assay

Tumor cells were plated in 96-well plate (Falcon 35–3075) in 180 μL growth media at 3000–5,000 cells/well. The cells were incubated overnight at 37 °C with 5 % CO_2_. On Day 2, dilutions of testing articles were added to the cell plate (20 μL/well) in triplicate wells. Untreated control wells (for 0 % control) and wells treated with 10 μM staurosporin (for 100 % kill) were included in each plate. The plates were incubated for 3–5 days at 37 °C with 5 % CO_2._ To quantify live cells, media was removed and 1x Cell Titer Aqueous One Solution (Promega, G3581) diluted in Opti-mem media (Invitrogen # 31985–070) was added to plates and the plates were then incubated for one hour at 37 °C. The OD at 490 nm was read on a M5 Spectramax plate reader (Molecular Probes). Percent inhibition was calculated based on 100 % kill and untreated control wells using the following formula: 100x (0 % control - treated)/(0 % control - 100 % kill). For cells grown in suspension such as SNU5, cells were plated in 96-well plate in 180 μL medium at 10,000 cells/well and incubated overnight at 37 °C with 5 % CO_2_. The same protocol as above was used for treatment and data processing except live cells were detected by adding forty μL of Cell Titer Aqueous One Solution to each well and the plates were then incubated for one hour at 37 °C. The experiments were repeated at least twice for each cell line.

### Anti-tumor efficacy studies in vivo

Studies with gastric (SNU5 and SNU620), lung (EBC1, NCI-H441) and glioblastoma (U87MG) were carried out at Pierre Fabre. Animal Ethical Committee was registered under the CEA-CIPF-108 number. All experiments conformed to the United Kingdom Co-ordinating Committee on Cancer Research (UKCCCR) Guidelines for the Welfare of Animals in Experimental Neoplasia UKCCR for animal care and use. SCID mice were from Charles River Laboratories (L’Arbresle, France). Athymic Nude Mice were acquired from Harlan (Gannat, France). All animals were housed on a 12 h light/dark cycle, in sterilized filter-topped cages, in a temperature 22 +/−2 °C and in humidity (30 to 70 %) controlled room. Mice were maintained in sterile conditions with food and water provided *ad libitum* and manipulated according to French and European guidelines. Animals were examined before the initiation of experiments to ensure that they were healthy and acclimated to the laboratory environment.

Cells (5–10 × 10^6^) were implanted s.c. into the right flank region of mice. Tumor bearing mice were size matched and randomized into study groups (*n* = 5 or 6 as shown in figure legends). Each experiment consisted of an ABT-700 dose evaluation injected i.p. compared to controls. Evaluation of the anti-tumor activity was determined by measuring tumor volume twice a week using the formula: π/6 × length × width × height.

For the ectopic EBC1 lung metastasis and survival model, mice were injected s.c. with 7x10^6^ EBC1 cells on D0, and after 5 days, when tumor volume reached 60 mm3 to 80 mm^3^, mice were size matched and randomized into groups (*n* = 7) for treatment with ABT-700 or control administered by i.p. injections every 21 days. From D5 to D21, tumor volume was monitored twice a week with an electronic caliper. On day 21, subcutaneous primary tumors were resected from mice anesthetized with a Ketamine/Xylazine mixture (70/30) injected intra-muscularly. ABT-700 antitumor activity was monitored by following animal mortality.

Additional animal groups were introduced for the gastric xenograft model SNU5 in order to evaluate pharmacodynamic markers by immunohistochemistry as described in detail in Additional file 2: Supplementary methods.

Experiments with the gastric Hs746T s.c. xenograft model were conducted at AbbVie in compliance with AbbVie’s Institutional Animal Care and Use Committee and the National Institutes of Health Guide for Care and Use of Laboratory Animals guidelines in a facility accredited by the Association for the Assessment and Accreditation of Laboratory Animal Care. SCID mice were obtained from Charles River (Wilmington, MA). Mice were acclimated to the animal facilities for a period of at least one week prior to commencement of experiments.

Hs746T cells (2 × 10^6^) were inoculated in the flanks of male SCID mice and tumor-bearing animals were size matched and randomly assigned to cohorts to a mean tumor volume of approximately 225 mm^3^ per group (*N* = 10) 15 days post inoculation of cells. Dosing for all agents was initiated on day 16. ABT-700 (10 mg/kg) was administered twice a week i.p. while docetaxel (7.5 mg/kg) was administered i.v. as a single dose. A human IgG control antibody was used as a negative control agent. Tumor dimensions were determined twice weekly and volume determined with the formula (L x W^2^)/2.

### Fluorescence in situ hybridization (FISH) analysis

MET gene copy numbers in cell lines, tumor xenografts and human tumor tissue specimens was detected with a probe mix (Vysis LSI MET SpectrumOrange/Vysis CEP 7 SpectrumGreen, Abbott Molecular) using protocols described in detail in Additional file 2: Supplementary methods.

### Statistical analysis

Results are expressed as the mean ± SEM. All data were analyzed with GraphPad Prism V6.05 (GraphPad Software, Inc., San Diego, CA). The difference in tumor growth between different groups was analyzed by two-way ANOVA Turkey’s multiple comparison tests. The survival data were analyzed using Log-rank (Mantel-Cox) test. A *P* value <0.05 was considered significant.

## Results

### Characterization of ABT-700 binding properties

ABT-700 is a humanized bivalent IgG1/kappa monoclonal antibody derived from the m224G11 mouse hybridoma [12]. ELISA and FACS-based assays were used to characterize the binding properties of ABT-700. In an ELISA format, ABT-700 binds recombinant human c-Met with an apparent affinity of 0.22 nM (Table 1). Similar binding to cynomolgus monkey derived c-Met was also observed although there was no detectable binding to mouse derived c-Met (not shown). These binding properties are similar to those observed for the parental mAb m224G11 [12].

**Table 1:** Binding affinity of ABT-700

	ABT-700 (nM)
Biding to c-Met protein by ELISA^a^	0.22
Binding to cellular c-Met by FACS^b^	
A549	0.23
IM95	0.24
SNU5	0.55
EBC1	0.24
MCF7	No binding
MCF7-human c-Met	1.3

^a^EC_50_ values were derived from an ELISA in which c-Met ECD was captured on the plate *via* a histidine tag. Value shown is averages of 6 experiments

^b^EC_50_ values were derived from FACS analysis of ABT-700 on various cell lines. Values are representative of at least two experiments

**Fig. 1:**
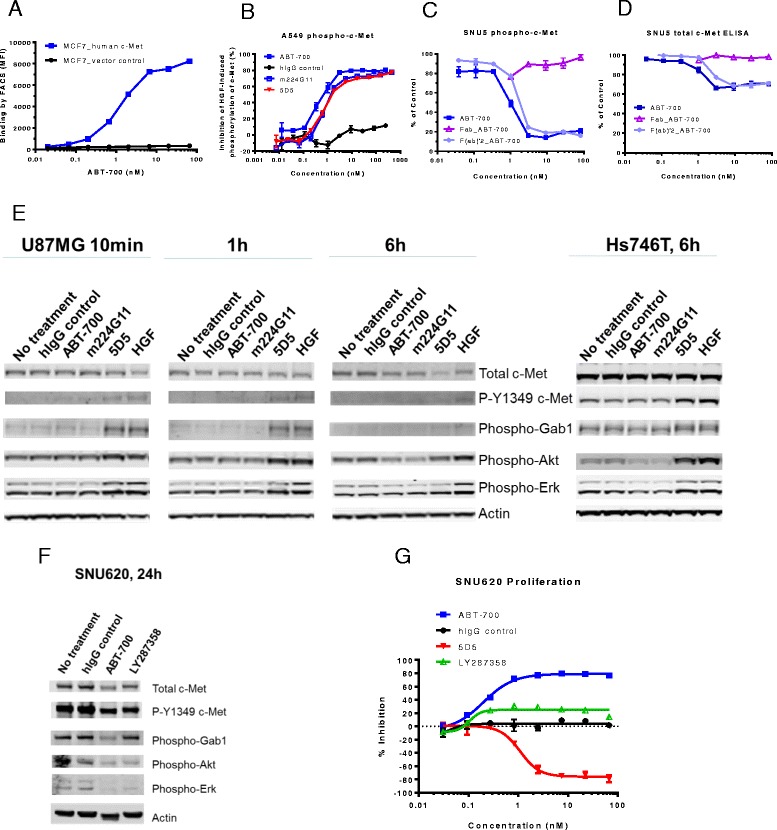
ABT-700 specifically binds cellular c-Met and antagonizes c-Met signaling in both HGF-dependent and -independent settings. **a** FACS analysis of ABT-700 binding to MCF7 transfectants. Stable human c-Met or vector control transfectants of human MCF7 breast cancer cells were incubated with increasing amounts of ABT-700 and bound ABT-700 was detected by FACS with secondary anti-human IgG conjugated with Alexa 488. **b** ELISA quantification of phospho-c-Met in A549 cells. A549 cells grown in a 96-well plate were pre-incubated for one hour with antibodies in a dose-range as shown, followed by stimulation with 1 nM HGF for 10 min. Total cell lysates were made and phospho-c-Met was detected by ELISA. **c** ELISA quantification of phospho-c-Met in SNU5 cells. SNU5 cells grown in a 96-well plate were incubated with antibodies in a dose-range as shown for 6 h. Total cell lysates were made and subjected to ELISA for phospho-c-Met. The value of cells in media alone was used as 100 % of control. **d** ELISA quantification of total c-Met in SNU5 cells. SNU5 cells grown in a 96-well plate were incubated with antibodies in a dose-range as shown for 6 h. Total cell lysates were made and c-Met level was determined by ELISA. The value of cells in media alone was used as 100 % of control. **e** Western blot analysis of U87MG cell lysates. U87MG cells grown in a 12-well plate were treated with antibodies as shown at 10 μg/mL for 10 min, 1 h or 6 h. Total cell lysates were analyzed for c-Met and other phosphorylated targets as shown. Western blot analysis of Hs746T cell lysates. Hs746T cells grown in a 12-well pate were treated with antibodies as shown at 10 μg/mL for 6 h. Total cell lysates were analyzed for c-Met and other phosphorylated targets as shown. **f** Western blot analysis of SNU620 cell lysates. SNU620 cells grown in a 12-well pate were treated with antibodies as shown at 10 μg/mL for 24 h. Total cell lysates were analyzed for c-Met and other phosphorylated targets as shown. **g** Inhibition of proliferation of SNU620 cells. SNU620 cells were plated in a 96-well plate and treated with antibodies in a dose range as shown for 3 days. Quantification of live cells at the end of incubation was done with Cell-titer Glo reagents. The data shown in all panels are from one of at least two independent experiments showing similar results as described in Methods

FACS analysis was used to compare the binding of ABT-700 to human tumor cell lines that express c-Met or harbor *MET* amplification. ABT-700 shows monophasic and saturable binding to cell lines expressing endogenous c-Met including A549, IM95, SNU5 and EBC1 with EC_50_ values of 0.2 – 1.3 nM (Table 1). There was no ABT-700 binding to the c-Met negative MCF7 breast cancer cell line, however following transfection to introduce endogenous c-Met expression into this cell line, significant ABT-700 binding was observed (Fig. 1a and Table 1). These results indicate specific and high affinity binding of ABT-700 to human c-Met.

### ABT-700 antagonizes c-Met signaling in both HGF-dependent and -independent settings

To determine the effect of ABT-700 on cellular c-Met phosphorylation induced by exogenous ligand HGF (1 nM) and/or by over-expressed c-Met in the absence of added HGF, we used ELISA-based assays to quantify phospho-c-Met in cell lysates of A549 and SNU5. ABT-700 and parental mAb m224G11 blocked the HGF-induced c-Met phosphorylation of A549 tumor cell line with an IC_50_ of 1.0 nM (Fig. 1b). 5D5, the parent murine monoclonal antibody of MetMAb [11], was also effective in blocking HGF-induced c-Met phosphorylation (Fig. 1b) although in the absence of HGF it can induce c-Met phosphorylation and agonistic activity (see section below). In contrast to the HGF-dependent A549 cell line, SNU5 gastric cancer cells express high levels of constitutively phosphorylated c-Met. ABT-700 treatment, but not 5D5 treatment (data not shown), caused more than 80% reduction of phosphorylated c-Met in SNU5 cells with an IC_50_ of 0.4 nM (Fig. 1c). ABT-700 also caused a decrease of total cellular c-Met by ~40 % in this cell line (Fig. 1d). Given that the decrease of c-Met phosphorylation was greater than that of c-Met protein, inhibition of phosphorylation and down-regulation of receptor may be caused through distinct mechanisms by ABT-700. The antagonistic activity of ABT-700 required the bivalent nature of the antibody because the Fab fragment did not inhibit c-Met phosphorylation or induce c-Met down-regulation (Fig. 1c and d).

Activation of c-Met tyrosine kinase induces phosphorylation of receptor adaptor proteins such as Gab1, triggering Akt and Erk signaling cascades [2, 3, 14]. Treatment with the agonistic c-Met antibody 5D5 mimics activity of the native ligand HGF that induces elevating levels of phosphorylated Gab1, Akt and Erk in both the HGF ligand-dependent U87MG and the *MET* constitutively activated Hs746T cells after treatment (Fig. 1e). In contrast, ABT-700 and its parental mouse hybridoma antibody m224G11 antagonize cellular c-Met signaling without causing agonist activity as evidenced by a decrease in phosphorylated signaling molecules including Gab1, Akt and Erk in both cell lines (Fig. 1e).

ABT-700 activity differs from the recently reported anti-c-Met antibody LY2875358 [15] in tumor cells where c-Met signaling can be driven by both receptor expression and HGF stimulation. SNU620 gastric cancer cells have high basal level of phosphorylated c-Met as shown in Fig. 1f, and their growth in vitro is stimulated by the agonistic anti-c-Met antibodies 5D5 (Fig. 1f). Similar effects on cell proliferation were also observed after treatment with HGF (data not shown). ABT-700 showed more robust inhibition of c-Met signaling and proliferation in SNU620 cells than LY2875358 although the latter was similarly effective (not shown) in other cellular models examined for ABT-700 as shown in Fig. 1. This difference was seen also in efficacy trials in vivo where ABT-700 but not LY2875358 inhibited the SNU620 tumor growth (described in sections below).

Taken together, these data suggest that ABT-700 is a differentiated anti-c-Met antibody with strong antagonistic effect on both HGF-independent constitutive c-Met signaling and HGF-dependent activation of c-Met.

### ABT-700 induces apoptosis in cell lines harboring *MET* amplification

**Fig. 2:**
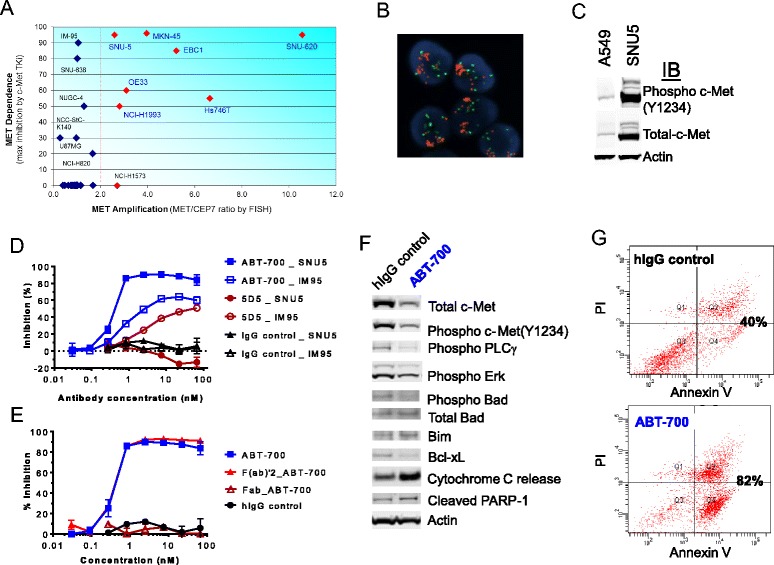
ABT-700 inhibits proliferation and induces apoptosis in *MET* amplified tumor cells. **a** *MET* status and dependence in a panel of 35 human cancer lines. Average of *MET/CEP7* was calculated in 20 random cells and the red symbol indicates cell lines harboring *MET* amplification (*MET/CEP 7* ratio ≥2). *MET* dependence was indicated by the maximal inhibition of proliferation of each cell line grown under regular medium containing FBS by the selective c-Met kinase inhibitor PF-4217903 at 1 μM for 3 days. Additional details are summarized in Table 1. **b** Image of FISH analysis of MET in SNU5 cells. Red represents signal of *MET* while green represents *CEP 7*. FISH images of additional cell lines are shown in Additional file 3: Figure S1. **c** Immune-blots of total and phosphorylated c-Met protein from total cell lysate of A549 and SNU5 cells as described in the legend of Fig. 1. **d** and **e** Inhibition of SNU5 proliferation. SNU5 cells were plated in 96-well plate and treated with antibodies or antibody fragments in a dose range as shown for 3 days. Quantification of live cells at the end of incubation was done with Cell-titer Glo reagents. Data are from one representative experiment. **f** Immune-blots of signaling and apoptosis pathway molecules in SNU5 cells treated with 10 μg/mL ABT-700 for 24h as described in the legend of Fig. 1. **g** Dual PI and Annexin V FACS analysis of SNU5 cells treated with 10 μg/mL ABT-700 for 24h. The percent of Annexin V positive apoptotic cells is shown. Data are from one experiment that was reproduced in independent experiments

To identify tumor lines with aberrant c-Met signaling suitable for further examination of the effect of ABT-700, we evaluated a panel of 35 human cancer cell lines for *MET* amplification, c-Met protein expression and their dependence on c-Met activity for growth in vitro. Most of the cell lines evaluated were derived from gastric cancer because of the high incidence of *MET* amplification associated with that cancer type [16, 17]. As summarized in Table 2 and Fig. 2a, we identified eight tumor cell lines exhibiting *MET* gene amplification as determined by fluorescent in situ hybridization (FISH) with an average ratio of *MET* and *CEP 7* (a centromere control) copy numbers of 2 or greater (Table 2 and Additional file 3: Figure S1). All cell lines harboring *MET* amplification except for NCI-H1573 showed dependence on c-Met signaling for growth in vitro (Table 2). NCI-H1573 cells do not have high level of c-Met protein despite possessing low level amplification of the *MET* gene (Table 2). On the other hand, there are cell lines that do not have MET amplification but show c-Met signaling dependence likely due to the presence of autocrine HGF (e.g. IM95) and/or c-Met overexpression (e.g. SNU638 and NCI-H820).

**Table 2:** Summary of MET status and sensitivity to ABT-700 in a panel of 35 human cancer lines

Cell line	Type	Source	FISH			WB		Proliferation	
			MET (mean/cell)	CEP7 (mean/cell)	Ratio MET/CEP7	Total c-Met^a^	Phospho-(Y1234) c-Met^a^	Max Inhibition by (%)	
								PF-4217903^b^	ABT-700^c^
SNU620	gastric	KCLB	41.6	4.4	10.6	6.7	56	95	80
H1993	lung	ATCC	32	11.9	2.8	5.4	829	50	30
Hs746T	gastric	ATCC	22.2	3.9	6.6	6	377	55	40
OE33	esophageal	Sigma	21.4	6.9	3.1	6	430	60	70
SNU5	gastric	ATCC	20.2	7.9	2.6	12	910	95	90
EBC1	lung	JCRB	15.6	3.1	5.2	6	374	85	30
MKN45	gastric	JCRB	12.6	3.4	4	10	297	96	50
H1573	lung	ATCC	11.5	4.3	2.7	1.8	5	0	0
H2342	lung	ATCC	8.6	5.2	1.7	0.7	1.3	0	0
H820	lung	ATCC	8.3	5	1.7	2.9	5.7	20	0
NUGC-2	gastric	JCRB	7.8	8.2	1	0.5	0.1	0	0
FU97	gastric	JCRB	7.6	7.5	1	0.1	0.1	0	0
NUGC-4	gastric	JCRB	6	5.1	1.3	1	15	50	0
SNU-16	gastric	ATCC	5.1	5.7	0.9	0.5	2.5	0	0
KATOIII	gastric	ATCC	4.6	5	0.9	0.9	16	0	0
SNU-216	gastric	KCLB	4.5	4.7	1	0.8	0.5	0	0
MKN-1	gastric	JCRB	4.4	3.8	1.2	0.2	0.1	0	0
SNU-484	gastric	KCLB	4.2	4	1	0.1	0.1	0	0
SNU-668	gastric	KCLB	3.6	3.6	1	1.9	0.5	0	0
SNU-1	gastric	ATCC	3.3	3.2	1	NA	NA	0	0
RERF-GC-1B	gastric	JCRB	3.1	4.2	0.8	0.6	0.6	0	0
OCUM-1	gastric	JCRB	3	2.9	1	0.4	0.3	0	0
SCH	gastric	JCRB	3	3.5	0.9	0.3	0	0	0
NCC-StC-K140	gastric	JCRB	2.8	9	0.3	0.5	0.3	30	0
SNU-719	gastric	KCLB	2.7	3.5	0.8	0.4	0.1	0	0
IM95	gastric	JCRB	2.5	2.5	1.1	0.2	0.5	90	60
MKN74	gastric	JCRB	2.4	2.6	1	0.1	0.1	0	0
SNU-601	gastric	KCLB	2.4	3	0.8	0.4	2.6	0	0
U87MG	glioblastoma	ATCC	2.1	2.1	1	0.5	3	30	30
23132/87	gastric	DSMZ	2	1.9	1.2	0.5	0.2	0	0
AGS	gastric	ATCC	2	2.2	1	0.2	0.1	0	0
NUGC-3	gastric	JCRB	2	3.2	0.7	0.5	1.1	0	0
SNU638	gastric	KCLB	2	2	1	3.6	28	80	50
TAKIGAW	gastric	JCRB	2	5.1	0.4	0.4	0.1	0	0
NCI-N87	gastric	ATCC	1	2	0.5	NA	NA	0	0
A549	lung	ATCC	NA	NA	NA	1	1	0	0

^a^Normalized to actin and relative A549 which was assigned 1

^b^at 1 μM concentration that is known to fully inhibit cellular c-Met with minimal off-target effect [21, 28]

^c^at 10 μg/mL (~67 nM) that saturates c-Met binding

The SNU5 gastric cancer cells harboring *MET* amplification and constitutively activated c-Met signaling was selected for further analysis to compare ABT-700 and antibody 5D5. The SNU5 cells contained an average of 20 copies of *MET* with a *MET/CEP 7* ratio of 2.6 (Fig. 2b and Table 2) and high levels of total and phosphorylated c-Met protein (Fig. 2c). The IM95 gastric cancer cell line was included for comparative analysis since it exhibits autocrine HGF signaling [18] and dependence on c-Met signaling in the absence of MET amplification (Fig. 2a and Additional file 3: Figure S1). ABT-700, but not 5D5, inhibited the proliferation of the *MET* amplified SNU5 cells, whereas both ABT-700 and 5D5 inhibited the growth of the HGF-driven IM95 cells (Fig. 2d). These results demonstrate that only ABT-700 blocked both amplification-driven (HGF-independent) and HGF-dependent c-Met activation. The antagonistic activity of ABT-700 against the constitutively activated c-Met requires bivalent binding because the Fab fragment of ABT-700 does not inhibit proliferation (Fig. 2e), consistent with the effects on c-Met phosphorylation observed in these cells (Fig. 1c).

Given that ABT-700 inhibited SNU5 cell growth by more than 90%, we further examined its effect on c-Met signaling pathways and induction of apoptosis in these cells. SNU5 cells possess constitutively activated c-Met as indicated by the high levels of phosphorylated c-Met (Fig. 2c and f). ABT-700 treatment down-regulated c-Met, and abrogated the level of phosphorylated c-Met and downstream signaling molecules including PLCγ, and Erk (Fig. 2f). ABT-700 treatment also activated the intrinsic apoptosis machinery resulting from the imbalance of pro- and anti-apoptotic proteins such as Bad and Bcl-xL. Phosphorylation of Bad leads to its ubiquitylation and degradation [19]. ABT-700 induced a decrease of phosphorylated Bad and the consequent stabilization of this pro-apoptotic protein, which was accompanied by elevation of the pro-apoptotic Bim and a decrease of the anti-apoptotic Bcl-xL (Fig. 2f). Other events consistent with the activation of the intrinsic apoptosis pathway were detected in the ABT-700 treated cells, including the release of cytochrome C into the cell cytoplasm and cleavage of the DNA repair enzyme, poly ADP ribose polymerase (PARP) (Fig. 2f). The increase in Annexin V staining of SNU5 cells following ABT-700 treatment further confirmed the occurrence of apoptosis (Fig. 2g). Furthermore, induction of apoptosis by ABT-700 was seen in other cell lines with amplified *MET* including SNU620 gastric cancer and EBC1 NSCLC but not in Hs746T and MKN45 gastric cancer cells (data not shown). Collectively, these results demonstrate that ABT-700 can inhibit the proliferation and consequently induce apoptotic cell death in cancer cells addicted to c-Met signaling driven by *MET* amplification.

### ABT-700 inhibits tumor growth in models driven by *MET* amplification

**Fig. 3:**
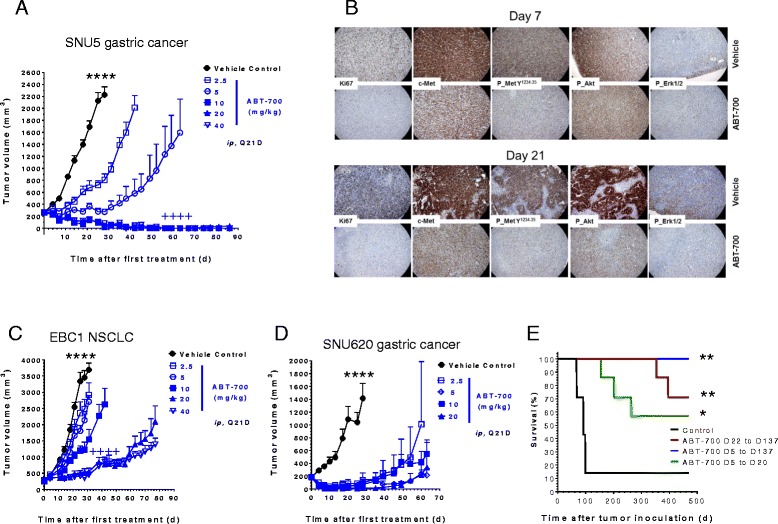
ABT-700 shows antitumor activity in preclinical models of tumor xenografts of human cancer cells harboring MET amplification. **a** Tumor growth curves of SNU5 gastric cancer treated with ABT-700. SCID mice with established tumors were treated with ABT-700 in a dose response administered by intra-peritoneal injections every 21 days. Each group had 6 mice; all ABT-700 treatment groups show significant difference (*P* value <0.0001 indicated by ****) when compared to the control group (day 4–28); high dose (10–40 mg/kg) groups are significant different from 5 mg/kg group (*P* value <0.0001 indicated by ++++) for day 4–63. **b** IHC analysis of SNU5 tumors treated with ABT-700. As in (**a**), tumors from mice treated with a single dose of ABT-700 at 10 mg/kg or vehicle control for 7 or 21 days were harvested and subjected to IHC analysis for markers as indicated. Representative images of one tumor are shown. **c** Tumor growth curves of EBC1 tumor xenograft model. SCID mice with established tumors were treated with ABT-700 in dose response administered by intra-peritoneal injections every 21 days. Each group had 5 mice; all ABT-700 treatment groups show significant difference (*P* value <0.0001 indicated by ****) when compared to the control group (day 4–31); high dose (20–40 mg/kg) groups are significant different from 10 mg/kg group (*P* value <0.0001 indicated by ++++) for day 4–42. **d** Tumor growth curves of SNU620 gastric cancer treated with ABT-700. SCID mice with established tumors were treated with ABT-700 in dose response administered by intra-peritoneal injections every 21 days. Each group had 6 mice; all ABT-700 treatment groups show significant difference (*P* value <0.0001 indicated by ****) when compared to the control group (day 4–28); there was no significant difference among treatment groups (day 4–28). **e** Survival curves of mice with metastatic EBC1 tumors treated with ABT-700. Primary tumors established after subcutaneous inoculation of EBC1 cells were surgically removed and ABT-700 at 10 mg/kg, Q21D, was administered with several schedules (start and end day) as shown. Growth of metastases in the lung caused death of animals and mortality was monitored over 530 days. Each group had 7 mice; comparison of survival curves by Log-rank (Mantel-Cox) test showed significance with *P* value <0.01 indicated by ** or <0.05 indicated by * as compared to the control group

Next we examined the effects of ABT-700 on tumor growth of subcutaneously implanted human tumor xenografts harboring amplified *MET*. ABT-700 inhibited the growth of established SNU5 tumors harboring amplified *MET* at doses ranging from 2.5 to 40 mg/kg, with long-term tumor regressions including complete responses observed at doses of ≥ 10 mg/kg (Fig. 3a). ABT-700 decreased both total and phosphorylated c-Met and the downstream signaling proteins, phosphorylated Akt and Erk, as measured by immunohistochemistry from treated tumor tissue collected on day 7 and/or 21 after a single dose at 10mg/kg (Fig. 3b). Cell proliferation in tumor tissue, indicated by Ki67 staining, was suppressed at both time points (Fig. 3b). These pharmacodynamic responses correlated with activity of inhibition of tumor growth; ABT-700 at doses below 5 mg/kg was not effective in inhibiting the tumor growth or the signaling proteins (Additional file 4: Figure S2). ABT-700 also inhibited the growth of four additional human tumor xenografts harboring *MET* amplification including EBC1 NSCLC and SNU620, Hs746T and MKN45 gastric cancer (Fig. 3c and 3d; and ref. [12]). Furthermore, ABT-700 was effective in prolonging survival in an EBC1 metastasis model (Fig. 3e). These results indicate that ABT-700 effectively antagonizes constitutively activated c-Met and downstream signaling leading to inhibition of tumor growth in preclinical tumor models with *MET* amplification.

**Fig. 4:**
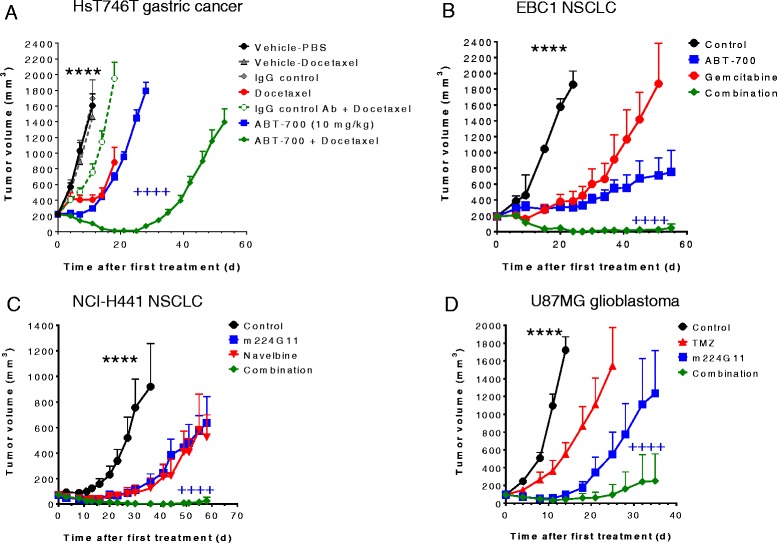
Combination of ABT-700 and chemotherapies exhibits enhanced antitumor activity in preclinical tumor models. **a** Tumor growth curves of Hs746T gastric cancer treated with ABT-700 in combination with docetaxel. ABT-700 at 10 mg/kg was administered twice a week for the duration of the experiment either alone or in combination. Docetaxel at a dose of 7.5 mg/kg was administered once at the start of dosing either alone or in combination. A human IgG control antibody was used as a negative control agent for ABT-700. Each group had 10 mice; there was no significant difference among the vehicle and isotype control groups; all treatment groups showed significant difference (*P* value <0.0001 indicated by ****) when compared to the control groups (day 4–11); there was a significant difference (*P* value <0.0001 indicated by ++++) between ABT-700 or docetaxel single agent and the combination group (day 4–28). **b** Tumor growth curves of EBC1 xenografts treated with ABT-700 in combination with gemcitabine. Athymic mice with established tumors were treated with ABT-700 at 20 mg/kg by i.p. injections every 21 days during the course of study either alone or in combination. Single dose of gemcitabine at 138.5 mg/kg was given on day 0 either alone or in combination. Each group had 5 mice; all treatment groups showed significant difference (*P* value <0.0001 indicated by ****) when compared to the control groups (day 6–24); there was a significant difference (*P* value <0.0001 indicated by ++++) between ABT-700 or gemcitabine single agent and the combination group (day 6–51). **c** Tumor growth curves of NCI-H441 NSCLC treated with m224G11 in combination with Navelbine. Athymic nude mice with established tumors were treated i.p. either with a loading dose of 2 mg of antibody/mouse and then twice a week with 1 mg of antibody/mouse until Day 33 either alone or in combination. Navelbine was dosed (on D0, D7, and D14) at 8 mg/kg by i.p. injections either alone or in combination. A third group administered with the combination treatment was also included. Each group had 6 mice; all treatment groups showed significant difference (*P* value <0.0001 indicated by ****) when compared to the control groups (day 3–36); there was a significant difference (*P* value <0.0001 indicated by ++++) between ABT-700 or navelbine single agent and the combination group (day 3–58). **d** Tumor growth curves of U87MG glioma treated with m224G11 in combination with TMZ (temozolomide). Athymic nude mice with established tumors were treated i.p. with a loading dose of 2 mg of antibody/mouse and then twice a week with 1 mg of antibody/mouse until Day 33 either alone or in combination. TMZ was given (on D0, D7, and D14) at 5 mg/kg by i.p. injections either alone or in combination. A third group administered with the combine treatment was included. Each group had 6 mice; all treatment groups showed significant difference (*P* value <0.0001 indicated by ****) when compared to the control groups (day 4–14); there was a significant difference (*P* value <0.0001 indicated by ++++) between ABT-700 or TMZ single agent and the combination group (day 4–35)

We also investigated whether ABT-700 in combination with established therapies would provide additional therapeutic benefit in preclinical animal models. In the *MET*-amplified Hs746T gastric cancer model, the combination of ABT-700 with docetaxel, an anti-mitotic microtubule inhibitor clinically used for gastric cancer, was more efficacious than either agent given as monotherapy (Fig. 4a). Although ABT-700 or docetaxel monotherapy at near maximal tolerated dose inhibited tumor growth in this model, the longer duration of response observed following combination therapy suggests that this combination may be a useful clinical strategy for delaying tumor relapse. A similar outcome was observed in NSCLC models where combination of ABT-700 and standard of care chemotherapeutics such as the nucleoside analog gemcitabine or the anti-mitotic drug vinorelbine (Navelbine) at near maximal tolerated doses. ABT-700 alone effectively suppressed the EBC1 tumor growth but was not able to induce complete responses (Figs. 3c and 4b). Single agent gemcitabine was also only partially effective (Fig. 4b). Combination of these two agents produced tumor regressions and eradications (Fig. 4b). In the NCI-H441 NSCLC xenograft model that is known to have overexpression of c-Met with gene amplification [20] (Fig. 4c), combination of the ABT-700 parental mAb m224G11 with Navelbine also resulted in more complete and durable control of tumor growth. U87MG is an aggressive glioma that possesses autocrine HGF c-Met signaling [21]. mAb m224G11 suppressed U87MG tumor growth demonstrating single agent activity against this HGF-dependent tumor (Fig. 4d). The current SOC therapy for GBM is radiation treatment in combination with the DNA alkylating agent, temozolomide (TMZ), although novel treaments that extend patient survival are urgently needed [22]. Addition of TMZ at near maximal tolerated dose to m224G11 therapy in the U87MG glioma xenograft model resulted in enhanced anti-tumor effects compared to either agent alone (Fig. 4d). Because neither the NCI-H441 nor the U87MG tumor models is *MET* amplified, these results suggest that ABT-700 in combination with existing therapies may broaden177 the clinical indications beyond those tumors with *MET* amplification to those tumors with c-Met overexpression, or autocrine HGF stimulation.

### *MET* amplification in gastric cancers and potential clinical utility of ABT-700

**Fig. 5:**
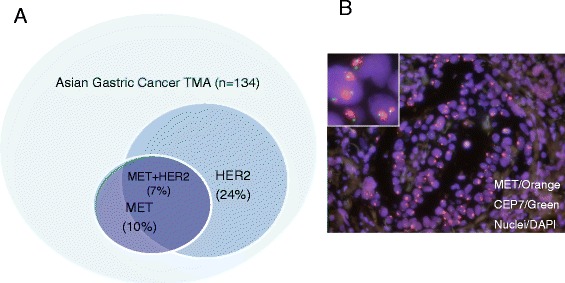
*MET* amplification as detected by FISH analysis in human gastric cancer specimens. **a** Venn diagram showing amplification frequency in a tissue microarray of gastric cancer samples of Asian patients. MET amplification was defined as average of MET/CEP 7 ratio ≥2 in chromosomally abnormal cells selected for enumeration. HER2 amplification was defined similarly but with the ratio of HER2/CEP 17 ≥ 2. **b** Composite image of a MET amplified gastric cancer sample. Insert shows clusters of amplified MET genes in the nuclei of tumor cells

*MET* gene amplification ranging from frequencies of 1-20% has been documented in a variety of human cancers, including liver metastases from colon carcinoma [23], non-small cell lung carcinomas with acquired resistance to EGFR inhibitors [24] and gastric cancers [16, 17]. A *MET* FISH assay was developed for detecting *MET* gene amplification in human biopsied cancer tissues utilizing a probe that spans 456 k-bases encompassing the entire *MET* gene located on the chromosome locus 7q31.2. This *MET* FISH assay was applied to a tissue microarray of 140 human gastric cancer samples obtained from Asian patients. As anti-HER2 antibody is indicated for the treatment of *HER2* positive gastric cancer, we also performed *HER2* FISH analysis. *MET* amplification as defined by *MET/CEP7* ratio ≥2 was seen in 10% of 134 evaluable patient samples, and 9 of the 13 *MET* amplified samples were also positive for *HER2* amplification (Fig. 5). This data suggest that patients with HER2 positive gastric cancers should not be excluded from treatment with c-Met inhibitors although further studies are required to confirm the co-existence of amplification of *MET* and *HER2* in this disease setting. Together with earlier studies [16, 17], the data suggest that *MET* amplification may represent a critical genetic aberration in gastric cancers and this patient population could benefit from treatment with ABT-700.

## Discussion

MET amplification is associated with poor prognosis in a variety of cancers highlighting the significant unmet medical need of this patient population. We describe preclinical results that justify the clinical investigation of ABT-700 in cancer types with addiction to the *MET* oncogene. In a phase 1 clinical trial in patients with advanced solid tumors [25], ABT-700 was well tolerated and the monotherapy at the recommended dose of 15 mg/kg demonstrated anti-tumor activity in patients with *MET* amplified solid tumors [26]. By RECIST, 3 of 5 patients with tumors harboring *MET* amplification, as determined by FISH, had a partial response (1 each ovarian, gastric and esophageal). Among these 3 patients, the duration of response was 19, 23, and 24 weeks, respectively [26]. This study has been expanded to enroll additional patients with *MET* amplification and to better define predictive biomarkers, safety and clinical benefit.

The tumor cell lines evaluated in our study herein that harbor *MET* gene amplification, as defined by FISH analysis, and overexpress c-Met protein, are sensitive to ABT-700. Cancer cells addicted to constitutively activated c-Met undergo apoptosis upon exposure to ABT-700 monotherapy leading to tumor regression in preclinical animal models. Our data emphasizes the need for a patient selection strategy that harbor *MET* amplification as they most likely would benefit from ABT-700 treatment. Ultimately, ABT-700 may have broader clinical utility as the prevalence of *MET* amplified tumors increases with disease progression, recurrence and/or treatment regimens [1–4]. Thus, acquiring fresh tumor biopsies for FISH analysis may be needed to identify patients with *MET* amplification. Recently somatic splice site alterations at *MET* exon 14 (METex14) that result in exon skipping and *MET* activation were described in human cancers [27]. Although we have not included METex14 analysis in our studies, it will be important to establish the clinical relevance of these genetic aberrations.

In addition to inhibiting tumors with MET gene amplification, ABT-700 also inhibits the subcutaneous xenograft growth of human tumor cell lines that have c-Met protein overexpression or autocrine HGF. Patients with tumors driven by HGF-dependent c-Met activation may benefit from combination of ABT-700 with chemotherapy. In this context, the demonstration of more robust and sustainable anti-tumor activity in animal models following the combination of ABT-700 with different standard of care cytoreductive chemotherapies provides the basis for exploration of effective combination therapy in the clinic.

ABT-700 is among the first reported bivalent anti-c-Met antibodies that lack agonistic activity [12, 15]. By inhibiting both ligand-dependent and c-Met overexpression-induced signaling in broad types of cancer cells, ABT-700 differentiates from other therapeutic agents targeting the HGF/c-Met axis [6, 7, 11] including the recently disclosed antagonistic IgG4 c-Met targeting antibody LY2875358 [15]. ABT-700 binds a unique epitope on c-Met outside the Sema domain which 5D5 recognizes [11, 12] and antagonizes c-Met signaling in a variety of cancer cells including SNU620 gastric cancers where LY2875358 demonstrated no in vivo antitumor activity. Since ABT-700 contains a humanized IgG1 heavy chain, antibody effector functions may also contribute to its antitumor activity. Results from in vitro ADCC functional assays indicate that ABT-700 can mediate the lysis of cellular targets by human natural killer cells (data not shown).

## Conclusions

ABT-700 shows preclinical activities in blocking both HGF-dependent and HGF-independent c-Met signaling, inducing apoptosis and suppressing tumor growth in cancers with amplified *MET.* Collectively, the attributes of ABT-700 coupled with early signs of activity in a phase 1 clinical trial support investigation of this antibody, as monotherapy and in combination, in malignancies characterized by aberrant activation of c-Met signaling including cancers harboring *MET* amplification.

## Additional files

Additional file 1: Table S1. Summary of tumor lines used in the study. (DOC 69 kb) Additional file 2:
**Supplementary Methods.** (DOC 29 kb) Additional file 3: Figure S1. FISH images of tumor cells. These signals correspond to two target loci/centromere on chromosome homologues to which each of the fluorescent probes are bound: green, CEP7; and orange, MET. (PPT 799 kb) Additional file 4: Figure S2. IHC analysis of SNU5 tumors treated with ABT-700 in dose response for 21. (PPT 1098 kb)

## Abbreviations

ABT-700: humanized monoclonal antibody against c-Met
5D5: mouse hybridoma antibody against c-Met
MetMAb: one-armed antibody derived from 5D5
FISH: fluorescence in situ hybridization
