# Supplementary material for: Anti-c-Met monoclonal antibody ABT-700 breaks oncogene addiction in tumors with MET amplification
Source: BMC Cancer. 2016 Feb 16;16:105. doi: 10.1186/s12885-016-2138-z (PMC4755020; doi:10.1186/s12885-016-2138-z)
Supplement: Additional file 1: Table S1. — Summary of tumor lines used in the study. (DOC 69 kb) [file 12885_2016_2138_MOESM1_ESM.doc]

**Additional file 1: Table S1. Summary of tumor lines used in the study**

| **Cell line** | **Type** | **Source** | **Catalog #** | **Culture media** |
| --- | --- | --- | --- | --- |
|
|
| SNU620 | gastric | KCLB | KCLB00620 | RPMI, 10% FBS |
| H1993 | lung | ATCC | CRL-5909 | RPMI, 10% FBS |
| Hs746T | gastric | ATCC | HTB-135 | DMEM, 10% FBS |
| OE33 | esophageal | Sigma | 96070808 | RPMI, 10% FBS |
| SNU5 | gastric | ATCC | CRL-5973 | RPMI, 10% FBS |
| EBC1 | lung | JCRB | JCRB0820 | DMEM, 10% FBS |
| MKN45 | gastric | JCRB | JCRB0254 | RPMI, 10% FBS |
| H1573 | lung | ATCC | CRL-5877 | RPMI, 10% FBS |
| H2342 | lung | ATCC | CRL-5941 | RPMI, 10% FBS |
| H820 | lung | ATCC | HTB-181 | RPMI, 10% FBS |
| NUGC-2 | gastric | JCRB | JCRB0821 | RPMI, 15% FBS |
| FU97 | gastric | JCRB | JCRB1074 | DMEM, 10% FBS, 10 mg/L Insulin |
| NUGC-4 | gastric | JCRB | JCRB0834 | RPMI, 10% FBS |
| SNU-16 | gastric | ATCC | CRL-5974 | RPMI, 10% FBS |
| KATOIII | gastric | ATCC | HTB-103 | DMEM, 20% FBS |
| SNU-216 | gastric | KCLB | KCLB00216 | RPMI, 10% FBS |
| MKN-1 | gastric | JCRB | JCRB0252 | RPMI, 10% FBS |
| SNU-484 | gastric | KCLB | KCLB00484 | RPMI, 10% FBS |
| SNU-668 | gastric | KCLB | KCLB00668 | RPMI, 10% FBS |
| SNU-1 | gastric | ATCC | CRL-5971 | RPMI, 10% FBS |
| RERF-GC-1B | gastric | JCRB | JCRB1009 | RPMI, 10% FBS |
| OCUM-1 | gastric | JCRB | JCRB0192 | DMEM, 10% FBS |
| SCH | gastric | JCRB | JCRB0251 | RPMI, 10% FBS |
| NCC-StC-K140 | gastric | JCRB | JCRB1228 | RPMI, 10% FBS |
| SNU-719 | gastric | KCLB | KCLB00719 | RPMI, 10% FBS |
| IM95 | gastric | JCRB | JCRB1075.0 | DMEM, 10% FBS, 10 mg/L Insulin |
| MKN74 | gastric | JCRB | JCRB0255 | RPMI, 10% FBS |
| SNU-601 | gastric | KCLB | KCLB00601 | RPMI, 10% FBS |
| U87MG | glioblastoma | ATCC | HTB-14 | DMEM, 10% FBS |
| 23132/87 | gastric | DSMZ | ACC-201 | RPMI, 10% FBS |
| AGS | gastric | ATCC | CRL-1739 | F12K, 10% FBS |
| NUGC-3 | gastric | JCRB | JCRB0822 | RPMI, 10% FBS |
| SNU638 | gastric | KCLB | KCLB00638 | RPMI, 10% FBS |
| TAKIGAW | gastric | JCRB | JCRB0124 | DMEM/RPMI, 10% FBS |
| NCI-N87 | gastric | ATCC | CRL-5822 | RPMI, 10% FBS |
| A549 | lung | ATCC | CCL-185 | DMEM, 10% FBS |
