# Supplementary material for: Anti-c-Met monoclonal antibody ABT-700 breaks oncogene addiction in tumors with MET amplification
Source: BMC Cancer. 2016 Feb 16;16:105. doi: 10.1186/s12885-016-2138-z (PMC4755020; doi:10.1186/s12885-016-2138-z)
Supplement: Additional file 2: — Supplementary Methods. (DOC 29 kb) [file 12885_2016_2138_MOESM2_ESM.doc]

**Additional file 2: Supplementary Methods**

**Immunohistochemistry staining:** For immunohistochemistry studies, tumors were removed after humane killing of mice using O_2_/ CO_2_ procedure (8 min cycle with Minerve equipment) at the determined points (n=3 for all groups, vehicle and ABT-700 treated animals at each dosage and each time). Tumor specimens were fixed overnight in 10% buffered formalin and subsequently embedded in paraffin. Five µm sections were cut and analyzed for the determination of proliferative index and for expression and phosphorylation levels of receptor.

Sections were rehydrated and antigen retrieval was done using S1699 retrieval buffer (Dako) for 40 minutes at 98°C. Endogenous peroxidases were quenched and sections were blocked at room temperature for 1 h with PBS/4%BSA. Sections were incubated with primary antibody and then incubated with horseradish peroxydase-conjugated polymer (Envision Dako) for 30 minutes at room temperature. Chromogenic signal was developed using 3,3’-diaminobenzidine (DAB Peroxidase Substrate Kit; Dako ). Tissues were counterstained with mayer's hematoxylin (Dako). Antibodies used for immunohistochemistry studies were incubated overnight at 4°C and included anti-phospho-c-Met (Abgent, San Diego, CA) ; anti-c-Met (Roche Ventana) at a 1:200 dilution ; anti-Ki67 at a 1:75 dilution (DakoCytomation, Glostrup, DN) ; anti-phospho Erk1/2 (Cell signaling, Danvers MA) at a 1/100 dilution and phosphor Akt (Epitomics, Burlingame, CA) 1/200 dilution.

**FISH procedures for cell lines**

Tumor cells were harvested from flasks when approximately 80% confluent and were cytospinned on to glass slides using a Shandon Cytospin III at 600 rpm, for 5 minutes. Cells were fixed in 1% methanol-free formaldehyde (Polyscience, Inc.) for 5 minutes, washed once with PBS and then once with water, and air dried. The slides were washed with 2X SSC at 73ºC for 2 minutes followed by incubation with 0.5mg/ml Pepsin (in 10mM HCl) at 37ºC for 10 minutes. After washing with 1X PBS at room temperature (RT) for 5 minutes, the cells were fixed sequentially with 1% NBF (Neutral-buffered formalin) at RT for 5 minutes, 1X PBS at RT for 5 minutes. The slides were dehydrated in a series of ethanol concentrations (70%, 85% and 100%) for one minute each and then air dried. After air drying, a 10 µl of probe mix (Vysis MET CDx FISH Kit (List #07N61-020)) was applied to the slide and covered with 18x18 mm coverslip. The slides were co-denatured in a ThermoBrite (Abbott Molecular, Inc.) at 73ºC for 3 minutes and hybridized at 37ºC overnight (a minimum of 16-20 hours). The slides were washed with 0.7X SSC with 0.3% NP-40 for 2 minutes at 73ºC and 2X SSC with 0.1% NP-40 for 1 minute at RT. After slides were air-dried, DAPI was applied. Slides were reviewed using fluorescence microscopy with Orange, Green and DAPI filters. Enumeration was carried out with the following procedure. Analysis started in the upper left quadrant of the targeted cells area. Fields were scanned from left to right and top to bottom, without re-scanning the same areas. Counterstain evaluation verified that the borders of nuclei observed by DAPI are distinguishable. Nuclei must have good integrity as evidenced by bright DAPI staining in order to perform the probe performance evaluation. Review each nucleus using the Orange specific, Green specific filters, before reviewing the next nucleus. Record signals patterns for the total of 25 nuclei in the target. Normal diploid nuclei are expected to exhibit four fluorescent signals (two green, and two orange). These signals correspond to two target loci/centromere on chromosome homologues to which each of the fluorescent probes are bound: green, CEP7; and orange, MET (7q31.2).

**FISH procedures for TMA**

Human gastric cancer TMA was purchased from Capital Biosciences. The TMA slides were baked in a ThermoBrite processor at 60ºC for approximately 4 hours. Deparaffinizing was done by immersing the slides in Hemo-De for 5 minutes at ambient temperature twice. The slides were dehydrated in 100% dehydrant for 1 minute at ambient temperature followed by pretreatment with 1X SSC at 80°C for 35 minutes and rinsed in purified water for 3 minutes at ambient temperature. The slides were treated with protease solution at 37°C for 10 minutes, washed with water. The slides were dehydrated in a series of ethanol concentrations (70%, 85% and 100%) for one minute each and then air dried. After air-dry, 20 µl of probe mix (Vysis MET CDx FISH Kit (List #07N61-020)) was applied to each target with 22x50 mm coverslip. The slides were then Codenature at 73ºC for 5 minutes on the ThermoBrite and then hybridized at 37ºC overnight (a minimum of 16-20 hours). After hybridization, the slides were washed with 2X SSC with 0.3% NP-40 for 3 minutes at 73ºC and 1X SSC for 1 minute at RT. After drying, DAPI was added and a coverslip applied. Slides were viewed using fluorescence microscopy with Orange, GREEN and DAPI filters. Enumerations were carried out with the following procedure. Begin enumeration of cells in the selected tissue spot using the prescribed orange and green filters and 60X to 63X objective. Choose tumor cells containing the highest MET copy number, count the number of orange signals and green signals. Record MET and CEP 7 counts in a worksheet. Some nuclei contain easily distinguishable MET orange signals, while others may contain MET signal clusters which represent localized aggregations of signals. When MET signal clusters are present, estimate the number of MET signals using increments of 5 or 10, depending upon the size of the cluster compared to a single signal. Enumerate each tissue spot in order to record 20 abnormal FISH cells. Stop the enumeration when 20 abnormal cells recorded. Otherwise, continue the enumeration until the whole tissue spot is examined. If cells with an increased MET copy number are not found, select the largest tumor cells per field of view to count the number of orange signals and green signals. Stop when 20 cells have been enumerated.

The same enumeration approach was applied to the slides hybridized with HER2 probe (•Vysis LSI HER-2/neu SpectrumOrange/CEP17 SpectrumGreen Probes).
