# Supplementary figures and images for: Anti-c-Met monoclonal antibody ABT-700 breaks oncogene addiction in tumors with MET amplification
Source: BMC Cancer. 2016 Feb 16;16:105. doi: 10.1186/s12885-016-2138-z (PMC4755020; doi:10.1186/s12885-016-2138-z)

## Slide 1
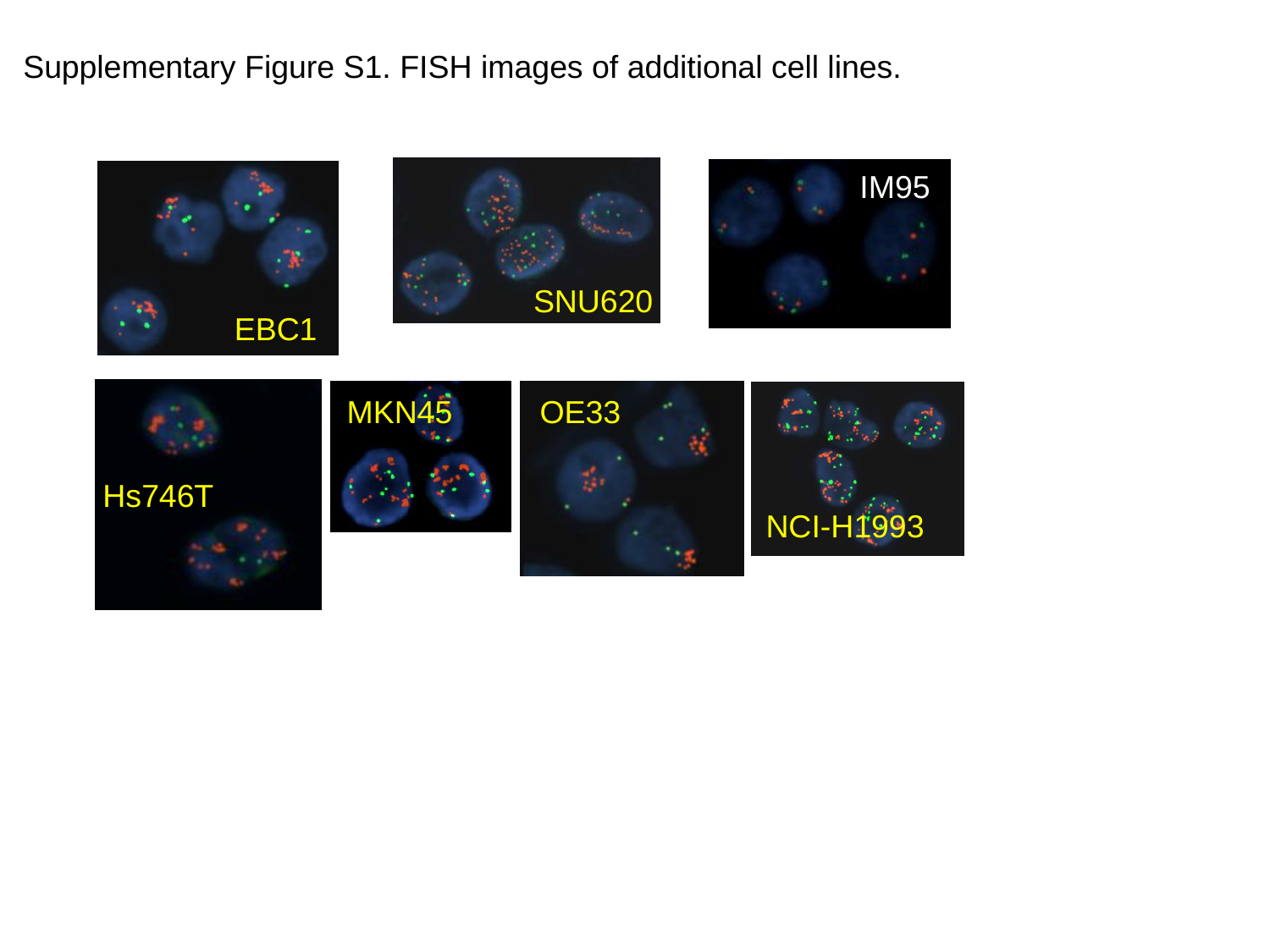

Supplementary Figure S1. FISH images of additional cell lines.
IM95
SNU620
EBC1
MKN45
OE33
Hs746T
NCI-H1993

Supplement: Additional file 3: Figure S1. — FISH images of tumor cells. These signals correspond to two target loci/centromere on chromosome homologues to which each of the fluorescent probes are bound: green, CEP7; and orange, MET. (PPT 799 kb) [file 12885_2016_2138_MOESM3_ESM.ppt]
