# Supplementary material for: Anti-c-Met monoclonal antibody ABT-700 breaks oncogene addiction in tumors with MET amplification
Source: BMC Cancer. 2016 Feb 16;16:105. doi: 10.1186/s12885-016-2138-z (PMC4755020; doi:10.1186/s12885-016-2138-z)
Supplement: Additional file 4: Figure S2. — IHC analysis of SNU5 tumors treated with ABT-700 in dose response for 21. (PPT 1098 kb) [file 12885_2016_2138_MOESM4_ESM.ppt]

## Slide 1
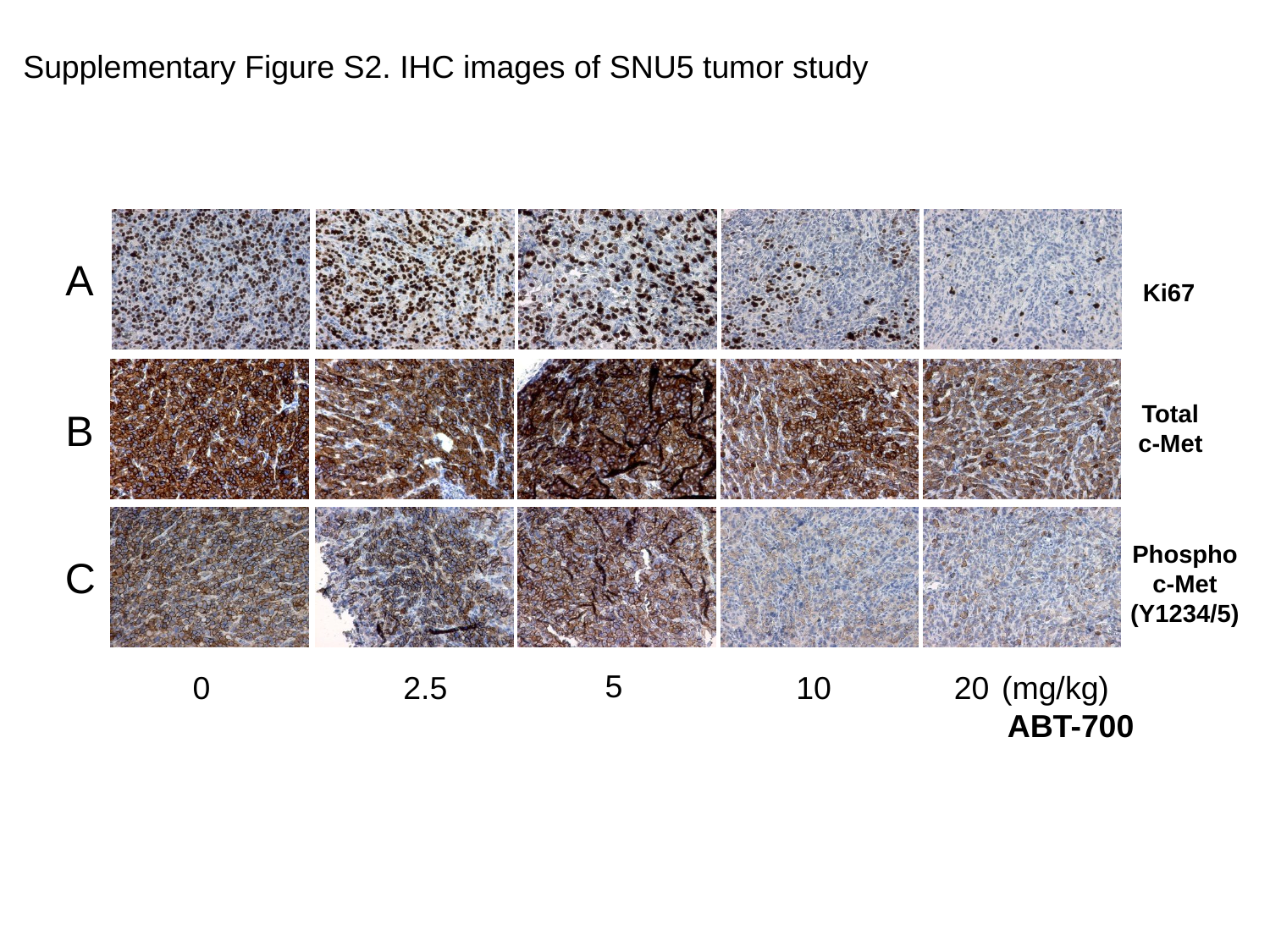

Supplementary Figure S2. IHC images of SNU5 tumor study
A
Ki67
Total c-Met
B
Phospho c-Met (Y1234/5)
C
5
0
2.5
10
(mg/kg)
 ABT-700
